# High azithromycin concentration in lungs by way of bovine serum albumin microspheres as targeted drug delivery: lung targeting efficiency in albino mice

**DOI:** 10.1186/s40199-016-0153-x

**Published:** 2016-05-05

**Authors:** Balakeshwa Ramaiah, Sree Harsha Nagaraja, Usha Ganganahalli Kapanigowda, Prakash Rao Boggarapu, Rajarajan Subramanian

**Affiliations:** Department of Pharmaceutics, Karnataka College of Pharmacy, #33/2, Tirumenahalli, Hegde Nagar Main Road, Bengaluru, Karnataka, 560064 India; Department of Pharmaceutical Sciences, College of Clinical Pharmacy, King Faisal University, Al-Ahsa, 31982 Saudi Arabia; Department of Pharmaceutical Technology, Karnataka College of Pharmacy, #33/2, Tirumenahalli, Hegde Nagar Main Road, Bengaluru, 560064 Karnataka India

**Keywords:** Azithromycin, Albumin microspheres, Sigma plot, Targeting efficacy, Lung targeting

## Abstract

**Background:**

Following administration, the antibiotic travels freely through the body and also accumulates in other parts apart from the infection site. High dosage and repeated ingestion of antibiotics in the treatment of pneumonia leads to undesirable effects and inappropriate disposition of the drug. By way of targeted lung delivery, this study was intended to eliminate inappropriate azithromycin disposition and to achieve higher azithromycin concentration to treat deeper airway infections.

**Methods:**

The Azithromycin Albumin Microspheres (AAM) was prepared by emulsion polymerization technique. The optimized AAM was subjected to in vitro release study, release kinetics, XRD and stability studies. Further, in vivo pharmacokinetics and tissue distribution of azithromycin released from AAM and azithromycin solution in albino mice was investigated to prove suitability of moving forward the next steps in the clinic.

**Results:**

The mean particle size of the optimized AAM was 10.02 μm, an optimal size to get deposited in the lungs by mechanical entrapment. The maximum encapsulation efficiency of 82.3 % was observed in this study. The release kinetic was significant and best fitted for Korsmeyer-Peppas model (R^2^ = 0.9962, *n* = 0.41). The XRD and stability study showed favorable results. Azithromycin concentration in mice lungs (40.62 μg g^−1^, 30 min) of AAM was appreciably higher than other tissues and plasma. In comparison with control, azithromycin concentration in lungs was 30.15 μg g^−1^ after 30 min. The azithromycin AUC (929.94 μg h mL^−1^) and intake rate (r_e_) (8.88) for lung were higher and statistically significant in AAM group. Compared with spleen and liver, the targeting efficacy (t_e_) in mice lung increased by a factor of 40.15 and ~14.10 respectively. Subsequently by a factor of 8.94, the ratio of peak concentration (C_e_) in lung was higher in AAM treated mice. The AAM lung tissue histopathology did not show any degenerative changes.

**Conclusions:**

High azithromycin concentration in albino mice lung was adequately achieved by targeted drug delivery.

## Background

Worldwide studies have revealed pneumonia to be the primary cause of death among children. Lung disorder generally affects 1 in 7 people and it is believed to be the major cause of death in the United States [1, 2]. The antimicrobial therapy has considerably reduced the outbreak of pneumonia, but there are instances reported on the failure of antimicrobial therapy. This was primarily owing to the increased antimicrobial-resistant pathogenic microorganisms, which resulted in the administration of ineffective treatments [3]. Following administration, drug travels freely through the body and also accumulates in other parts apart from the infection site. High dosage and repeated ingestion of antibiotics in the treatment of pneumonia leads to undesirable effects and inappropriate disposition of the drug.

The sub-therapeutic antibiotic concentration at the infection site and prolong usage of antibiotic has led to antibiotic resistance [4]. These issues facilitate the requirement of a targeted drug delivery model to maximize the therapeutic efficacy against pneumonia. Drug targeting at the site of infection minimizes dosage of the drug required to achieve the optimum serum concentration leading to obtain the desired pharmacological action. The role of drug targeting was found to be evident considering various parameters such as pharmaceutical, biopharmaceutical, pharmacokinetic, pharmacodynamic and clinical [5]. Additionally, development of targeted drug for treatment of pneumonia improves health care and promises survival of the patient [1].

Azithromycin, (9-deoxo-9a-aza-9a-methyl-9a-homoerythromycin A) dihydrate, a macrolide antiobiotic used as a broad spectrum antibiotic was proved to be efficacious in the treatment of pneumonia. It is primarily active against *Haemophilus influenza, Moraxella catarrhalis, Streptococcus pneumonia, Chlamydophila (Chlamydia) pneumonia* and *Mycoplasma pneumonia* [6]. Azithromycin has high affinity towards the 50S ribosomal subunit of the organism and blocks its protein synthesis. Several unique pharmacokinetic properties such as excellent tissue penetration, high volume of distribution and prolong half life has lead the use of azithromycin for the treatment of pneumonia. Azithromycin has very large volume of distribution (2100 L) results in good tissue penetration and a long half-life (40–60 h) [4]. The antimicrobial activity of azithromycin against pneumonia pathogens depends on its relative concentration at the infection site. Therefore increasing localized azithromycin tissue concentration will clear the pathogen more rapidly and reduce the possibility of drug resistance.

The tissue minimum inhibitory concentration (MIC_90_) breakpoint of azithromycin susceptibility against major organisms in US citizens was found to be ≤4 μg mL^−1^ [6]. As though to support this theory, clinical studies [7, 8] showed the azithromycin coverage in lungs was high with extended release of single or double dose microspheres. The better dosage regimen has improved azithromycin concentration especially in lower respiratory tract. The only novel marketed azithromycin microsphere (Zmax in USA) had proven to increase the plasma concentration by two to threefold to that of the conventional preparations [4]. The antimicrobial and post antibiotic effect (PAE) of azithromycin are characterized as concentration dependent [9]. By drug targeting, azithromycin concentration at the site of infection will be higher when compared to serum [6]. On intravenous administration, microspheres with 7–15 μm particle range are capable of being entrapped by lung capillaries [10].

In the recent past, a great deal of awareness has been observed in the preparation of microspheres using albumin for targeted drug delivery. The rationale was to specifically deliver the drug to the target tissues or cells by evading other tissue from undesirable effect [11]. A drastic increase, in the use of albumin in drug delivery was observed since 1970s proving albumin as a drug carrier and depicting greater attention in drug delivery system [12]. Albumin microspheres are nontoxic, physically and chemically stable, moreover they have specific receptor affinity in lungs. Albumin has numerous binding sites for exogenous ligands such as antibiotics and the properties of acidic, solubility, stability in pH range of 4–9 and preferential uptake by inflamed cells allows it to be chosen as ideal polymer. Bovine Serum Albumin (BSA) microspheres by phagocytosis are rapidly removed from the vascular system. On digestion of the albumin by lysosomal enzymes leads to release of free drug. Various drugs such as streptomycin, ofloxacin, clarithromycin and sodium cromoglicate when used with albumin microspheres, has significantly proven to be efficacious in lung targeting [13].

The microspheres intended as targeted delivery, injected via intravenous route enables to treat the deep lung disorders from the vascular side eliminating the need to traverse the mucus/surfactant and mucosal layers. At situations of compromised lung capacity and inhalation is not a viable option, use of injecting drug via intravenous route would be particularly helpful. It is evident that higher drug disposition in lungs and prolonged retention may translate into reduced doses, less frequent administration and lower bioavailability variability. Hence, the main purpose of this study was to prepare, develop and in vivo characterization of lung targeting efficiency to achieve high azithromycin concentration in lungs by way of Azithromycin Albumin Microsphere (AAM) as targeted drug delivery. The excellent tissue penetration enables azithromycin to get inappropriately deposit in various tissues such as liver and spleen apart from the infected lungs. Thus, the minimization of inappropriate azithromycin disposition and maintenance of higher azithromycin concentration in lungs can effectively combat the deeper lung tissue infections.

## Methods

### Materials

Azithromycin was acquired as a gift sample from Karnataka Antibiotic Pharmaceutical Limited, Bengaluru, India. BSA fraction V was purchased from SD Fine-CHEM limited, Mumbai, India. The Tween 80, heavy liquid paraffin, n-hexane, glutaraldehyde, petroleum ether, sodium bisulphite and Span 80 were obtained from Merck Specialties Pvt. Ltd, India. All the ingredients were of analytical grade.

### Experimental design

The central composite design was used to optimize azithromycin loaded albumin microspheres (AAM) by altering albumin, glutaraldehyde and Span 80 concentrations. The independent variable factors effect on mean particle size of the microspheres along with encapsulation efficiency and 6^th^ hour in vitro drug release was investigated. The model contained eight factorial points, six axial points and six centre points with total 20 experiments. The mean value was set as 0 and, +1 and −1 was considered as higher and lower levels for each factor respectively. The selected factor constraints with their levels along with optimized levels are summarized in Tables 1 and 2.

**Table 1:** Variable factors with their levels used for optimization of AAM

Variable factors	Level					Optimized level
	−1.41	−1	0	1	1.41	
Albumin concentration (%)	16.59	20	25	30	33.41	22.24
Glutaraldehyde volume (%)	0.16	0.3	0.50	0.7	0.84	0.58
Span 80 volume (mL)	0.20	0.4	0.70	1.0	1.20	1.0

**Table 2:** Response factor constraints with expected and observed values for optimized AAM

Response factors	Constraints	Expected value	Observed value	Residual value
Mean particle size (μm)	Target of 8 μm	9.04	10.02	0.98
Encapsulation efficiency (%)	In range	81.4	80.00	–1.4
6^th^ hour in vitro drug release (%)	In range	77.27	78.00	0.73

### Preparation of AAM intended for lung targeting

Azithromycin microspheres were prepared using albumin, a biodegradable polymer by emulsion polymerization technique [14]. Initially, BSA was dissolved in distilled water (22.24 %) and 0.5 mL of Tween 80 was added to the aqueous phase. Secondly, finely triturated 250 mg of azithromycin was added into the aqueous phase with bath sonication (3 min) for consistent dispersion. While stirring at 2500 rpm, 1 mL of the above solution was slowly incorporated in to 30 mL of heavy liquid paraffin containing a lipophillic surfactant, Span 80. The mixture was homogenized for 10 min to form water/oil emulsion. To crosslink the albumin, the emulsion was homogenized by adding glutaraldehyde. Further, the emulsion was homogenized for 5 min with addition of 5 mL n-hexane for hardening the formed microspheres. The microspheres were then centrifuged at 1000 rpm at room temperature for 1 min and washed with petroleum ether to remove the heavy liquid paraffin. To remove the residual glutaraldehyde, the microspheres were dispersed in 10 mL of 5 % w/v sodium bisulphite solution and stirred on a magnetic stirrer for 5 min. The microspheres were filtered through Whatmann filter paper with pore size of 0.45 μm and again washed with 100 mL of water to completely remove residual glutaraldehyde. At room temperature, the microspheres were dried and were stored in a dessicator.

### Particle size measurement of the optimized AAM

The particle size analysis [15] was carried out by photon correlation spectroscopy in Malvern Meta sizer, UK (He-Ne laser beam at wavelength of 633 nm and 90^0^ scattering angle). Ten milligram of the preparation was dispersed in 0.1 % of tween 80 containing 10 mL of water and was subjected to mean particle size determination. The mean particle size was averaged after three reading of the samples. The PCS software (Malvern Instruments Inc.) was used for processing of the data.

### Estimation of azithromycin

Reverse phase high performance liquid chromatography (RP-HPLC) was used as analytical tool for quantitative estimation of azithromycin [16]. Stainless steel column (25 cm × 4.6 mm) with end-capped octadecylsilyl amorphous organosilica polymer, 5 μm (Waters Xterra) was used for analysis. Mobile phase in the ratio of 40:60 consisted of 0.18 % w/v anhydrous disodium hydrogen phosphate solution (pH adjusted to 8.9) and a mixture of methanol (25 volumes) with acetonitrile (75 volumes), respectively. The injection volume was 50 μL with flow rate of 1 mL/min. At 60 ^0^C column temperature, UV detector was set at 210 nm. The retention time of the drug peak was observed at 5.916 min.

### Estimation of drug encapsulation efficiency of the optimized AAM

The encapsulation efficiency was estimated by solvent extraction method [11]. Ten milligram of the microspheres was dispersed in 0.5 mL of methylene dichloride. Later, 4.5 mL of ethanol was added to precipitate the polymer. After stirring at 14,500 rpm for 10 min, the supernatant was separated and diluted with 10 times by PBS (pH 6.8, 50 mM). By previously mentioned RP-HPLC, the quantification of the drug was performed. Equations (1) and (2) were utilized to determine the drug encapsulation efficiency.

1 $$ Drug\  loading\ \left(\%\right)=\frac{D_t}{M_t} \times 100 $$

2 $$ Encapsulation\  efficiency\ \left(\%\right)=\frac{L_a}{L_t} \times 100 $$

*D*_*t*_: amount of encapsulated drug in microspheres; *M*_*t*_: microsphere quantity; *L*_*a*_: actual drug content and *L*_*t*_: theoretical drug content.

### X-ray diffraction (XRD) analysis

The XRD analysis of azithromycin powder, BSA and AAM formulation (Pre and post stability studies at refrigeration and accelerated conditions) was performed using Philips X’Pert Pro, Netherlands at 40 KV voltages and applied 30 mA of current (nickel-filtered CuKα radiation). The sample was scanned over a 2θ range of 10–800 with an interval of 0.020 at the rate of 20/min.

### In vitro study and curve fitting analysis of the optimized AAM

Azithromycin release from AAM was estimated using Franz diffusion cells. The cell consisted of a dialysis membrane with MW cut-off of 12,000–14,000 Da (Himedia Laboratories Pvt. Ltd, Mumbai, India). This membrane acted as a barrier between donor and acceptor compartment. Hundred milligram of the microspheres dispersed in 1 mL of phosphate saline buffer (PBS) was placed in the donor compartment. The PBS was prepared by dissolving 0.2 g of potassium chloride, 1.44 g of disodium hydrogen phosphate, and 0.24 g of dihydrogen potassium phosphate in 800 mL of distilled water. The pH was maintained at 7.4 and the volume was made upto 1000 mL [17]. At 37 ± 0.5 ^0^C, 200 mL of PBS was placed in the acceptor compartment and stirred on a magnetic stirrer at 200 rpm. At specified time, 1 mL of the solution was pipetted out from the acceptor compartment and was replaced with the same volume of PBS. The drug content was determined utilizing the formerly mentioned RP-HPLC method. After three runs, the data was utilized to determine release kinetics.

Using Sigma plot, the release kinetics of the optimized AAM was fitted to various models such as first order model (3), Higuchi square root model (4), Baker and Lonsdale (5), Koresmeyer-Peppas (6) and Hixson and Crowell cube root model (7) to study the release of drug from the microspheres.

3 $$ Log\ \left(100-{Q}_t\right)= \log {Q}_0-\frac{K_t}{2.303} $$

4 $$ {Q}_t = {k}_H\sqrt{t} $$

5 $$ \frac{Q_t}{Q_{\infty }}=1 - \frac{6}{\pi^2} \exp \left(\frac{-{\pi}^2 \times Dt}{r^2}\right) $$

6 $$ {Q}_t = {Q}_0 + a{\left(\frac{t}{r^2}\right)}^n + b{\left(\frac{t}{r^2}\right)}^{2n} $$

7 $$ \sqrt[3]{Q_0} - \sqrt[3]{Q_t}={\mathrm{k}}_{\mathrm{HC}}t $$

*Q*_*t*_ is the total amount of drug release after *t* time (%); Q_0_ is the initial amount of drug (%); *K* is the first order release rate constant (h^−1^); k_H_ is the rate constant obtained according to the Higuchi equation (%h^–1/2^); Q_∞_ is the percent release at infinite time; *D* is the diffusion coefficient in the polymer in cm^2^/*s; r* is the radius of the sphere in cm; *n* and *2n* are the release exponent for Fickian diffusion and case II transport, respectively; *a* and *b* are constants related to the drug and the structural and geometric properties of the microparticles; and k_HC_ is the rate constant obtained according to the Hixon and Crowell equation (%h^−1^) [18–20].

### Stability study of the optimized AAM

The stability study was conducted as per ICH Q1AR guideline, intended to test the stability for new substances and product. The optimized preparation was stored at 5 ± 3 ^0^C for twelve months and at 25 ± 2 ^0^C and 60 ± 5 % RH for a period of six months. The required volume of microsphere dispersion was stored in closed glass bottles and sealed tightly. At regular intervals, the sample was subjected for determination of encapsulation efficiency, mean particle size distribution and for any physical changes. The test was carried at 3 month intervals for a period of 12 months for long term storage condition under refrigeration and at 0, 2, 4 and 6 months for accelerated condition at room temperature. To confirm the stability of the drug in the formulation, the samples were also subjected to XRD analysis.

### In vivo pharmacokinetic studies in albino mice

The animal experiments were carried out in accordance to the protocol approved by the Institutional Animal Ethics Committee formed under CPCSEA guidelines. Prior to the study, thirty six adult male and female, Swiss Albino mice, weighing 20 ± 3 g was kept starving for 12 h with free access to water. AAM microspheres were dispersed in saline with 1 % of Tween 80 and vortexed for 5 s. The control group received azithromycin (in saline) solution containing a dose of 50 μg g^−1^ injected intravenously via tail vein, while the AAM group was injected with equivalent content of azithromycin in AAM.

### Blood or organ isolation and extraction

At specified intervals (0.5 h, 1 h, 3 h, 6 h, 8 h and 12 h), the blood samples were collected from the ocular artery directly from each mouse after eye ball removal and placed into heparinized test tubes. By centrifugation (4000 g) for 15 min, the plasma was immediately separated. The animals were then sacrificed by cervical dislocation. The plasma and tissue samples of targeted organs such as lung, liver and spleen were isolated and stored at –20 ^0^C for 24 h. To determine the targeted release, the azithromycin concentration in each organ was determined by subjecting 1 g (after removal of surface water) of the tissue sample through extraction process. The normal saline (0.1 mg mL^−1^) was added to the isolated organs and homogenized. To precipitate protein, 2 mol L^−1^ perchloric acid (100 μL) was added to the plasma sample and homogenized tissues. Subsequently, 300 μL of plasma or homogenized tissues suspension was added to 450 μL methanol. The mixture was vortexed for 60 s and then centrifuged at 10,000 g for 5 min [17]. The supernatant was collected and filtered through a 0.22 μm pore size Phenomenex filter. The azithromycin concentration in the processed samples was quantitatively analyzed by above mentioned RP-HPLC method.

### Pharmacokinetic parameters

Change in azithromycin concentration with time was monitored in blood, lung, liver and spleen. Based on the analysis of parameters and model, the two compartment model could best describe the in vivo pharmacokinetics of microspheres in blood. The azithromycin, area under the curve (AUC_0–∞_) was determined from the beginning of the intravenous injection (t_0_) to the last observation (t_last_) by linear trapezoidal rule with extrapolation to infinite time. Additionally, azithromycin half life (t_1/2_ α and β), distribution rate constants (K_21_, K_10_ and K_12_), clearance (CL) and apparent volume of distribution at steady state (V_SS_) were also calculated. The Kinetica 5.0 PK/PD analysis software was also utilized for the calculation of pharmacokinetic parameters.

### Lung targeting characteristics and histopathological studies

The lung targeting efficiency of AAM was evaluated by using intake rate (r_e_), targeting efficacy (t_e_) and peak concentration ratio (C_e_) [21]. At the end of the study (12 h post sample administration), the animals were subjected to experimenting process by excess anesthesia. To examine the tissue tolerability of the formulation specified organs (lungs, liver and spleen) were washed with cold saline. Later, the organs were pressed between filter pads and weighed. Using 10 % formalin, the tissues were fixed and stained with hematoxylin and eosin. Under light microscopy with 200 × magnification, the tissue samples were examined for any cytoarchitecture changes.

### Statistical analysis

Statistical analysis was carried out by using Student’s *t* test with a *P* value less than 0.05 was considered as statistically significant.

## Results and discussion

### Preparation of AAM

Compared to other macrolides, azithromycin enhances extended spectrum and potency especially against pneumonia [5]. The biocompatibility, low toxicity, non antigenicity and physicochemical property of the BSA influenced to select it as an ideal polymer in this study. The preparation of albumin microsphere involved suspending an aqueous solution of albumin in an external non-polar phase. The nuclei formation due to the micronized azithromycin induces small droplet formation during emulsification resulting in smaller microsphere until steady state droplet size distribution. Glutaraldehyde a five-carbon dialdehyde, chemically cross links with albumin lysines. The glutaraldehyde as a fixative enhances covalent stabilization of the albumin producing stable and biocompatible microspheres. However, the residual glutaraldehyde could be toxic and was removed by bisulphate wash. The carbonyl group pi bond of glutaraldehyde was subjected to nucleophilic addition of bisulphate resulting in organic sulphite (water soluble sodium salt), which was further removed by water washing. Many earlier studies [22, 23] have successfully used emulsion polymerization for albumin microsphere preparations. This method was found to be advantageous, as the microspheres from the high molecular weight polymers are usually shaped at a faster rate and at low temperature.

### Mean particle size and drug encapsulation efficiency

Albumin microspheres in the range of 1–100 μm influence its biodistribution characteristics. Particle size was found to be a significant factor to distinguish between soluble carrier and particulate systems. The particle size being an important parameter for drug targeting with microspheres, as the size helps in discharge of drug in controlled manner and uptake of drugs into the tissues. In this study, the particle size ranged from 3.9–19.8 μm and the mean particle size of the optimized AAM was 10.02 μm. Various other studies [17, 22, 24] have reported similar particle size for albumin microspheres. Following intravenous administration, 7–15 μm particle size range of microspheres gets deposited in the lungs by mechanical entrapment [10]. To support this hypothesis, Kutscher et al. [25] proved that 10 μm sized microspheres were uniformly distributed throughout the lung capillaries to that of 3 μm sized microspheres, which usually pass through the lungs.

The injected microspheres primarily get lodged in alveolar capillaries that have diameters considerably less than those of the microspheres and thus, the concept of mechanical entrapment offers a unique opportunity for passive targeting of microspheres to lungs. Since there is a possibility of larger microspheres occluding larger capillaries leading to blockage of small downstream vessels may result in augmentation of potential toxic effect. To resolve this issue and avoid embolism due to injected microspheres, the USP has stated that “>90 % of microspheres must have a size between 10–90 μm and no microspheres may be larger than 150 μm”. A study by Glenny et al. [26] reported that the blood flow in rat lungs was not significantly affected by 15 μm particle size of the microspheres and neither emboli nor tissue infarction was observed. The same study states that these microspheres will occlude only a small fraction of capillaries (0.5–0.7 %) and significantly do not alter local vascular resistance. Within the capillaries, red blood cells can continue to flow past lodged microspheres.

In fact, the lung at rest only uses approximately 30 % of its capacity and is therefore able to recruit other capillaries to avoid massive increases in the arterial pressure when blockages do occur. Additionally, the lung utilizes variety of ways such as recruiting unused vessels and use of arteriovenous shunts to improve the blood circulation in the pulmonary capillaries. Microspheres with 1–3 μm particle size passes through the lung and will be removed by reticuloendothelial system and, usually accumulate in liver and spleen, excrete through the feces or sequestration by macrophages in other organ systems [25].

The maximum encapsulation efficiency of 82.3 % was observed in this study. The high encapsulation efficiency may also have contributed to low partition coefficient of azithromycin. Studies done by Bozdag et al. [24] and Jones et al. [27] stated that the albumin microspheres can be sustained and the encapsulation efficiency can be increased by escalating glutaraldehyde volume. The swelling, encapsulation efficiency and drug release profile can be controlled by cross linking density.

### In vitro study and curve fitting analysis

**Fig. 1:**
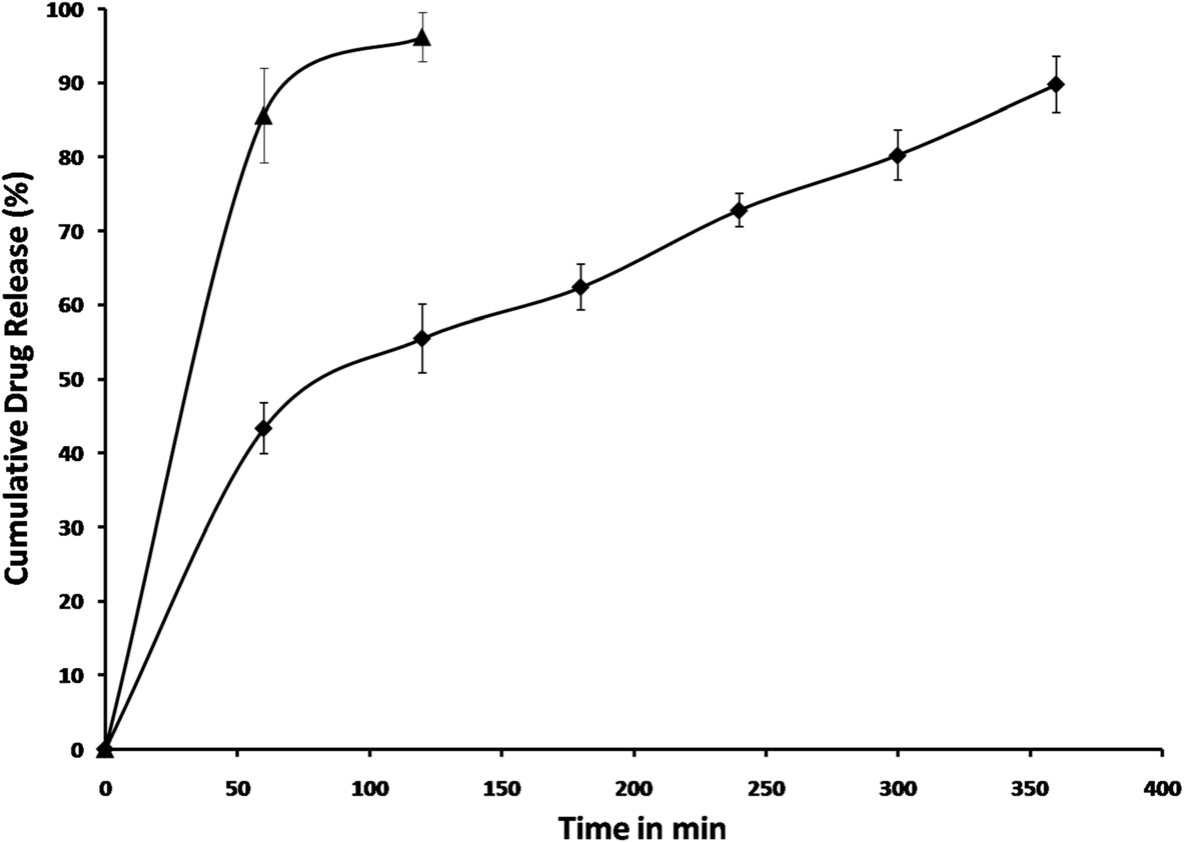
Cumulative amount of drug released () optimized AAM and () azithromycin solution (bars represent mean ± SD; *n* = 3)

AAM in vitro release study (Fig. 1) showed a minimum of 40.34 % and a maximum of 94.63 % of azithromycin release at the end of 1^st^ hour and 6^th^ hour, respectively. AAM showed biphasic in vitro drug release viz, an initial burst followed by sustained release at the end. The initial burst was due to the dispersed drug close to the microsphere surface and additionally, high encapsulation also contributes to this effect. The diffusion of dispersed drug in the polymeric matrix and BSA erosion would lead to the sustained release of the microspheres. Initial burst effect was required to provide the loading dose of the drug to combat the high bacterial load which usually seen during the initial phase of pneumonia.

**Fig. 2:**
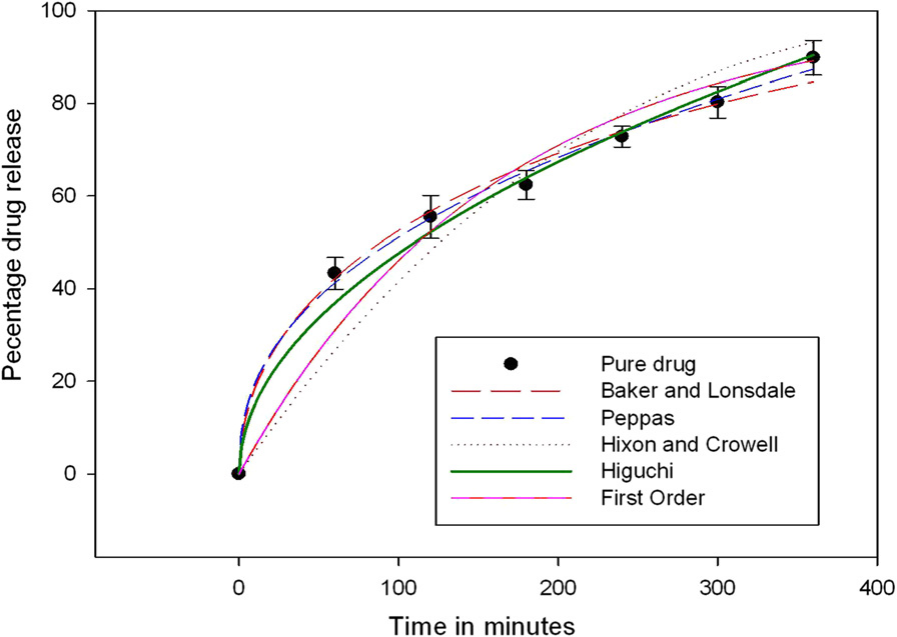
In vitro release profile of the optimized AAM-curve fitting models (bars represent mean ± SD; *n* = 3)

The release pattern of the optimized formulation on sigma plot has been represented in Fig. 2. The regression coefficient of Korsmeyer-Peppas model (R^2^ = 0.9962) was significant. The Korsmeyer-Peppas model illustrates the drug release mechanism from polymeric devices. To describe the drug release process, the *n*-value can be obtained by fitting data into Korsemeyer Peppas model. The *n*-value was found to be 0.41, indicating Fickian diffusion type of release. Due to dispersion of the azithromycin in polymeric matrix, the dissolution may not be rate limiting step, but the diffusion of the drug through polymeric matrix considered to be the slowest step for drug release. A study [14] showed similar behavior for albumin microspheres.

### XRD analysis

**Fig. 3:**
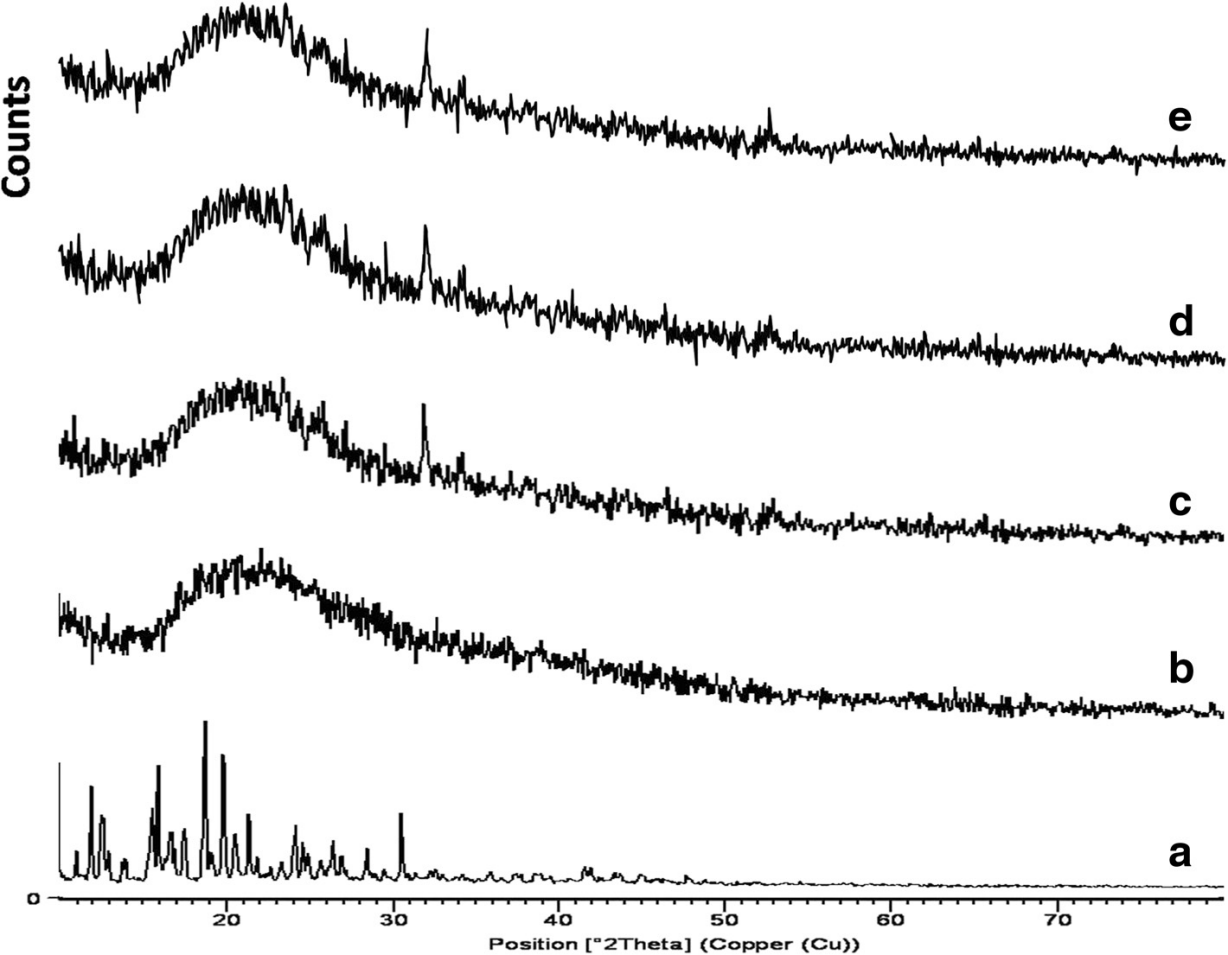
XRD of (**a**) azithromycin; (**b**) BSA; (**c**) optimized AAM before stability study; (**d**) optimized AAM after refrigeration (5 ± 3 °C) for 12 months; (**e**) optimized AAM after acceleration (25 ± 2 °C) for 6 months

XRD of the pure azithromycin, albumin and AAM (Pre and post stability studies at refrigeration and accelerated conditions) are shown in Fig. 3. XRD spectral characteristic of the azithromycin pure drug shows many diffraction peaks, indicating the crystallinity of the drug. In contrast, the diffraction peaks was significantly reduced in AAM. XRD of BSA shows one peak, which indicates non crystallinity. The AAM formulation showed decreased crystallinity of azithromycin, which was similar to that of BSA indicating the incorporation of azithromycin in the polymer.

### Stability study

The stability test observations of the optimized AAM at room temperature and refrigeration conditions are depicted in Tables 3 and 4. On storage, no major deviations were observed in the macroscopic characteristics. There were no changes in mean particle size of the optimized formulations stored at 25 ^0^C. On storage, the extent of microsphere sedimentation was not prominent, on manual agitation they were redispersed easily. Even though, there was slight decrease in the encapsulation efficiency (3.1 %), but was within the acceptable range. Thus, the optimized formula proved to be stable on long term and accelerated storage conditions as well.

**Table 3:** Stability test observations of the optimized AAM at refrigeration for long term storage condition

Storage	Encapsulation efficiency (%)					Mean particle size (μm)					Physical change				
5 ± 3^0^C	Months					Months					Months				
	0	3	6	9	12	0	3	6	9	12	0	3	6	9	12
	68.1	67.5	66.8	66.0	65.0	10.02	09.98	10.01	10.02	10.02	--	--	--	--	--

--: No physical change

**Table 4:** Stability test observations of the optimized AAM at room temperature for accelerated condition

Storage	Encapsulation efficiency (%)				Mean particle size (μm)				Physical change			
25 ± 2^0^C	Months				Months				Months			
	0	2	4	6	0	2	4	6	0	2	4	6
	68.1	67.8	67.3	66.9	10.02	10.02	10.01	10.01	--	--	--	--

--: No physical change

### In vivo pharmacokinetic studies

Based on the analysis of parameters and model, the two compartment model could best describe the in vivo pharmacokinetics of microspheres in blood. The pharmacokinetic parameters are illustrated in Table 5. The decisive parameters for the penetration into biological fluids and tissues are the drug molecular weight, lipophilicity and protein binding [13]. Compared to control, AAM altered in vivo azithromycin distribution and the half-life of azithromycin released from AAM intravenous injection (t_1/2_ (α) = 0.950 h, t_1/2_(β) = 11.38 h) were prominently higher than the intravenous injection of azithromycin solution (t_1/2_ (α) = 0.522 h, t_1/2_(β) = 10.32 h). This data proves the sustained release efficacy of AAM. Azithromycin concentration in mice lungs (40.62 μg g^−1^, 30 min) of AAM was appreciably higher than other tissues and plasma. In comparison with control, azithromycin concentration in lungs was 30.15 μg g^−1^ after 30 min. A clinical study [28] has shown that the initial high upfront release of azithromycin from the microspheres helps in achieving higher azithromycin concentration required to act against the early bacterial burden at the infection site in lungs.

**Table 5:** Pharmacokinetic parameters after intravenous injection of AAM and azithromycin solution (control) in albino mice

Pharmacokinetic Parameters	AAM	Azithromycin solution
AUC_0–∞_ (μg h mL^−1^)	32.73	88.70
t_1/2_ (α) (h)	0.950	0.522
t_1/2_ (β) (h)	11.38	10.32
K_21_ (h^−1^)	0.400	0.940
K_10_ (h^−1^)	0.111	0.940
K_12_ (h^−1^)	1.88	0.360
CL (h^−1^)	0.102	0.061
V_ss_ (L)	9.49	1.26
C_0_ (μg mL^−1^)	6	11

Data are represented as means ± SD (*n* = 3)

**Fig. 4:**
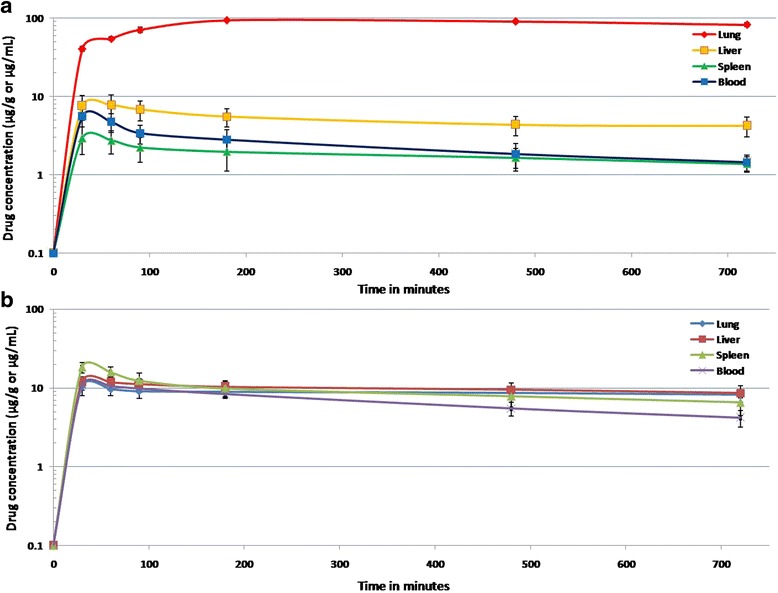
(**a**) Semilogarithmic plot showing azithromycin distribution in mice after intravenous injection of AAM (bars represent mean ± SD; *n* = 3); (**b**) Semilogarithmic plot showing azithromycin distribution in mice after intravenous injection of azithromycin solution (bars represent mean ± SD; *n* = 3)

After intravenous injection of AAM and azithromycin solution preparations, the distribution of azithromycin with time was estimated in lung, liver, spleen (μg mL^−1^) and blood (μg g^−1^). Azithromycin concentration in blood and other organs was considered as 100 %. The targeting parameters are shown in Table 6. The semilogarithmic plot showing azithromycin distribution in mice after intravenous injection of AAM and azithromycin solution (control) are shown in Fig. 4a and b, respectively. Azithromycin tissue distribution was found to be higher than plasma concentration in all time points. Azithromycin concentration in lungs considered to be a vital factor in achieving an effective clinical treatment. The capillary blockade (as a function of particle size) resulting in mechanical filtration leads to accumulation of microspheres in lung.

**Table 6:** Lung-targeting parameters after intravenous administration of AAM and azithromycin solution (control) in albino mice

Parameters	AUC^a^		r_e_	t_e_		(t_e_)_AAM_/(t_e_)_Control_	C_p_^a^		C_e_
	AAM	Control		AAM	Control		AAM	Control	
Blood	32.73	88.69	0.369	28.42	1.18	24.06	5.60	10.80	0.519
Liver	65.97	120.45	0.547	14.10	0.870	16.21	7.75	12.00	0.646
Spleen	23.16	121.01	0.191	40.15	0.866	46.39	2.94	18.27	0.160
Lung	929.94	104.75	8.88	1.00	1.00	1.00	93.65	10.47	8.94

r_e_ = (AUC)_AAM_/(AUC)_Control_

t_e_ = (AUC)_lung targeted_/(AUC)_untargeted_

C_p_: peak concentration (μg mL^−1^or μg g^−1^)

C_e_ = (C_p_)_AAM_/(C_p_)_Control_

^a^unit of AUC: μg h mL^−1^ or μg h g^−1^

AAM showed the highest value of AUC (929.94 μg h mL^−1^) and re (8.88) for lung, and the difference was statistically significant (*p = 0.0011*). The AAM targeting efficacy (t_e_) of lung increased by a factor of 40.15 (compared with spleen) and ~14.10 (compared with liver). The targeting ratio of AAM increased by a factor of 46.39 (compared to spleen) and ~16.21 (compared to liver). Additionally, compared with control the ratio of peak concentration in lung (C_e_) increased by a factor of 8.94. Literatures state that PAEs are usually investigated at ten times the MIC and in this study the azithromycin concentration was all time maintained above MIC. Among macrolides, the longer retain time (t > MIC) at the target site and strongest PAE up to 2.3–4.7 h of azithromycin results in relatively short treatment period [9].

Albumin microsphere improves circulatory half-lives of the drug by inhibiting drug uptake by reticuloendothelial system [14]. In this study, the albumin microsphere certainly has further influenced the accumulation of azithromycin particularly in lungs. The presence of specific albumin binding protein in alveolar epithelial cells further increases affinity to albumin leading to higher azithromycin concentration especially in lungs [29]. The protection of microspheres from opsonization and decreased urinary clearance of BSA (67 kDa) considered to be also a beneficial effect [30]. High azithromycin loading in lung tissues by AAM compared to control confirms the alteration in biodistribution of the azithromycin thus, the lung targeting characteristic of AAM was evident.

**Fig. 5:**
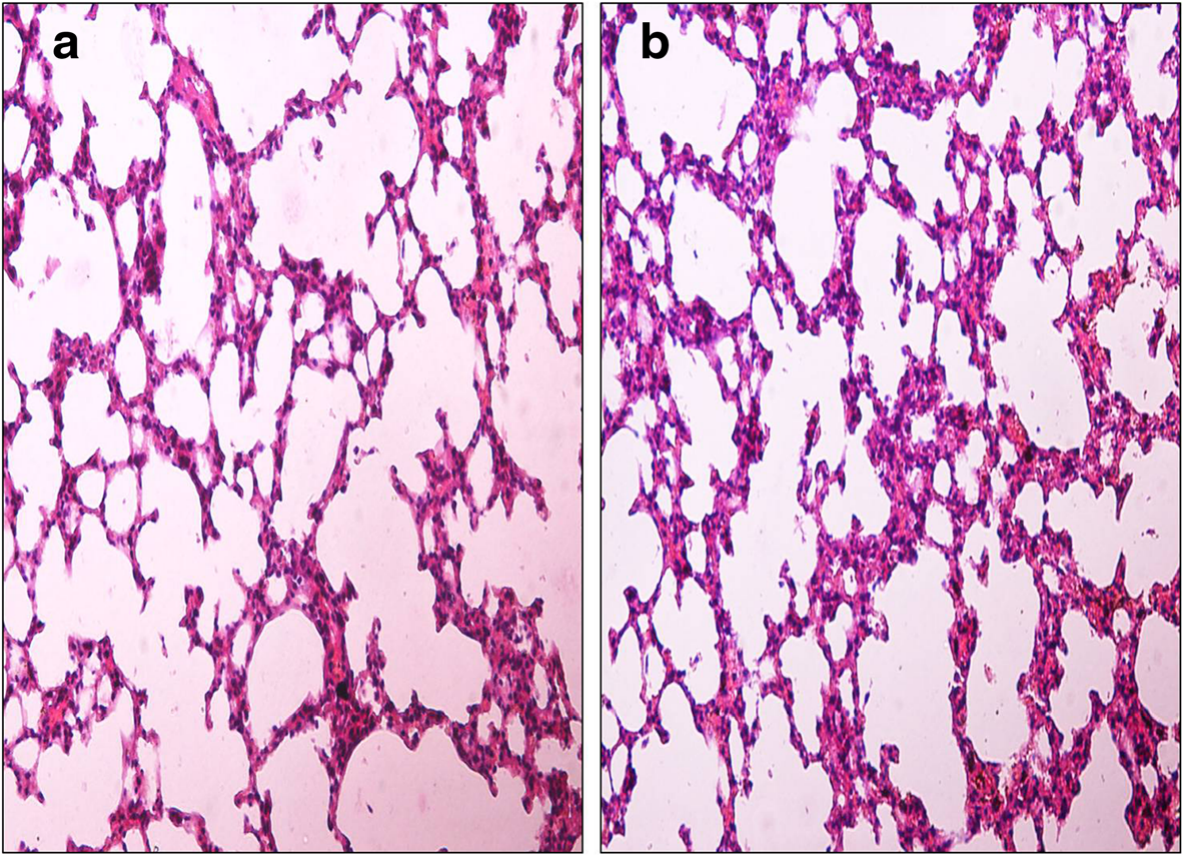
(**a**) Cytoarchitecture of albino mice lung (*azithromycin solution*); (**b**) Cytoarchitecture of albino mice lung (AAM)

The tissue tolerability considered to be a major concern as in microsphere based targeted system due to accumulation of the drug and excipients in the targeted organs. The histopathology of the mice lungs (Fig. 5a and b) did not show any degenerative changes in the AAM formulation compared to control group. This proves the safety and biocompatibility of the AAM as a parenteral formulation for lung targeting.

## Conclusions

Currently a single dose extended release azithromycin formulation has been found to be the only FDA approved antibiotic for the treatment of pneumonia. This study successfully formulated azithromycin incorporation into albumin microspheres. The particle size, encapsulation efficiency, in vitro study, release kinetics, XRD and stability study showed the suitability of the microspheres for lung targeting. In comparison with control, AAM showed better azithromycin concentration in lungs, higher AUC, the ratio of peak concentration (C_e_) and intake rate (r_e_). The favorable in vivo pharmacokinetics, lung targeting efficacy and histopathology proved applicability of the microspheres as targeted drug delivery to lungs.
